# Controlled Stimulus-Responsive Delivery Systems for Osteoarthritis Treatment

**DOI:** 10.3390/ijms252111799

**Published:** 2024-11-02

**Authors:** Qianwen Ye, Mingshuo Zhang, Shuyue Li, Wenyue Liu, Chunming Xu, Yumei Li, Renjian Xie

**Affiliations:** 1 School of Medical Information Engineering, Gannan Medical University, Ganzhou 341000, China; yeqianwen@gmu.edu.cn (Q.Y.); zhangmingshuo@gmu.edu.cn (M.Z.); lishuyue@gmu.edu.cn (S.L.); liuwenyue@gmu.edu.cn (W.L.); 2 Jiangxi Provincial Key Laboratory of Tissue Engineering (2024SSY06291), Gannan Medical University, Ganzhou 341000, China; xcm7604@gmu.edu.cn; 3 School of Basic Medicine, Gannan Medical University, Ganzhou 341000, China; 4 Key Laboratory of Prevention and Treatment of Cardiovascular and Cerebrovascular Diseases (Ministry of Education), Gannan Medical University, Ganzhou 341000, China

**Keywords:** osteoarthritis, drug delivery system, nanomedicine, stimulus response, cartilage regeneration

## Abstract

Osteoarthritis (OA), a common and disabling degenerative joint disease, affects millions of people worldwide and imposes a considerable burden on patients and society due to its high prevalence and economic costs. The pathogenesis of OA is closely related to the progressive degradation of articular cartilage and the accompany inflammation; however, articular cartilage itself cannot heal and modulate the inflammation due to the lack of nerves, blood vessels, and lymph-vessels. Therefore, reliable and effective methods to treat OA remain highly desired. Local administration of drugs or bioactive materials by intra-articular injection of the delivery system represents a promising approach to treat OA, especially considering the prolonged joint retention, cartilage or chondrocytes targeting, and stimuli-responsive release to achieve precision OA therapy. This article summarizes and discusses the advances in the currently used delivery systems (nanoparticle, hydrogel, liposome, and microsphere) and then focuses on their applications in OA treatment from the perspective of endogenous stimulus (redox reactions, pH, enzymes, and temperature) and exogenous stimulus (near-infrared, magnetic, and ultrasound)-responsive release. Finally, the challenges and potential future directions for the development of nano-delivery systems are summarized.

## 1. Introduction

**Figure 1:**
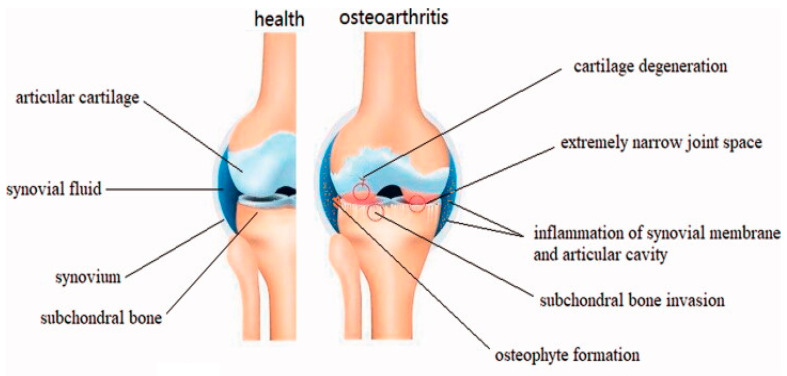
Schematic representation of healthy knee joint structures and pathological changes in knee OA. The erosion of articular cartilage begins on the surface and then gradually deepens into the calcified cartilage area. Chondrocytes (as the only cell type within articular cartilage) attempt to repair the erosion by enhancing their proliferation and differentiation; however, the accompanying inflammation significantly inhibits the function of chondrocytes, and therefore, the erosion of articular cartilage gradually develops until the subchondral bone is exposed. Simultaneously, the formation of osteophytes around the joint margins could be observed due to the endochondral pathologic enhancement of osteogenesis. Reprinted with permission from Ref. [10], copyright 2021, Mao et al.

Articular cartilage, also known as hyaline cartilage, cushions the end of bones in diarthrosis, and its main functions are to facilitate the load transmission and maintain the frictionless articulation of bones under high pressure during individual lifetimes [1]. However, articular cartilage is a tissue without blood vessels, nerves, and lymphatic vessels, leading to its very limited self-regenerating capacity [2,3,4]. Therefore, cartilage defects caused by trauma always fail to heal by themselves and progressively initiate the irreversible development of osteoarthritis (OA) [5,6]. Furthermore, the etiology of OA is multifaceted and predominantly influenced by a variety of additional factors, such as occupation (such as athletes with overuse of joints), age and gender (affecting about 9.6% of men and approximately 18% of women over 60 years old), obesity (increasing the burden of joints), physical activity (with the rising life expectancy to exercise), and heredity [7,8,9]. OA is a chronic disease characterized by progressive articular cartilage degeneration and concomitant synovium inflammation, and its main pathological changes are manifested by thinning of the subchondral bone, formation of periarticular bony encumbrances, meniscus changes, ligament damage, and hypertrophy of the joint capsule (Figure 1) [10,11]. Currently, knee OA has affected about 5% of population in the world, and the incidence and prevalence of OA are gradually increasing with the aging population and increasing obesity rates [11,12]. Once OA occurs, inflammatory cytokines (such as interleukin (IL)-1β, IL-6, IL-8, and tumor necrosis factor alpha (TNF-α)) and matrix metalloproteinases (MMPs) are overexpressed, and the levels are further upregulated within the progression of OA to accelerate cartilage degradation [8,13,14,15]; therefore, OA was recognized as a “serious disease” by the Food and Drug Administration (FDA) in 2022 [16]. Notably, no effective reliable method is available to identify individuals with OA before there are symptomatic or treatments are required [17,18,19]. Indeed, developing new treatment options for OA is always highly desired.

At present, pharmacological and surgical options are the conventional approaches for OA treatment [20,21]. However, pharmacological treatments (such as oral administration of analgesic or anti-inflammatory drugs and articular injection of glucocorticoids or hyaluronic acid (HA)) are primarily focused on pain relief and anti-inflammation, and the clinical outcomes are significantly limited by the potential side effects (gastrointestinal and cardiovascular complications and renal failure and infection due to highly frequent articular injection) [6,22], as well as the poor cartilage-targeted adsorption or accumulation at the OA cartilage site due to the highly hydrophobic common anti-inflammatory drugs and dense cartilage matrix networks [6,23]. Surgical treatments (such as microfracture, autologous chondrocyte implantation or matrix-induced autologous chondrocyte implantation, and joint replacement) are limited by the regeneration of fibrocartilage, scarce sources of donors or chondrocytes, the situation of large-area cartilage damage or late OA, and the risk of second surgery [24]. Therefore, the current options or OA treatment are far from curative and thus are largely palliative, especially considering most of them cannot reverse the progression of OA [25,26].

**Figure 2:**
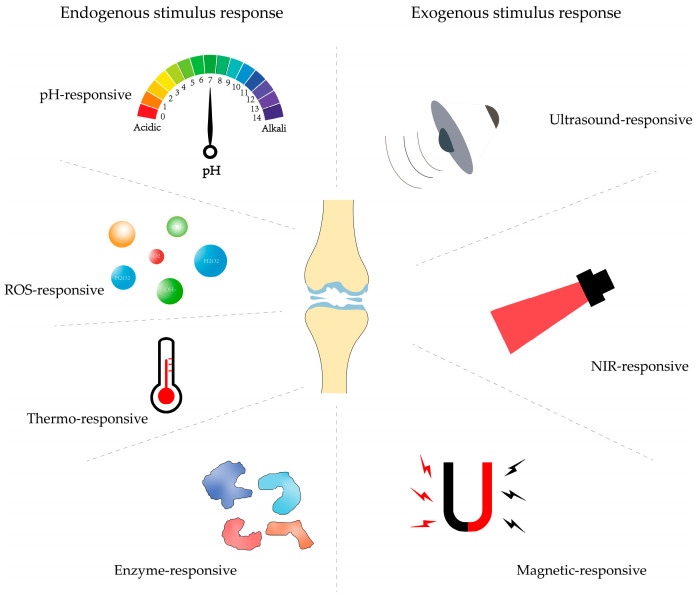
Stimulus-responsive delivery systems for articular cartilage repair and regeneration. The delivery systems are generally administrated to OA-affected joints via articular injections; thereafter, the drugs aimed to alleviate the OA conditions could be released from the delivery systems in response to the environmental stimuli, which contain endogenous stimuli and exogenous stimuli. The endogenous stimuli mainly include pH, reactive oxygen species (ROS), temperature, and enzymes. Correspondingly, the exogenous stimuli are ultrasound, near-infrared (NIR) therapy, and the magnetic field.

Over the recent years, considerable advances of drug delivery systems (DDSs) have been achieved, which present their robust potential to evade the disadvantages of conventional OA treatments [27], since the DDSs can be directly administrated to synovial joints by articular injection, and then, their properties can be finely tailored to achieve OA treatment outcomes [28]. For instance, stimulus-responsive nanocarriers in DDSs typically have multiple environmentally sensitive functions and are therefore capable of responding to multiple environmental stimuli, which include endogenous stimuli such as pH, redox potential, enzymes, and temperature, as well as exogenous stimuli such as electromagnetism, light, and ultrasound, to achieve precise releases of drug-carrying therapeutic agents [29,30]. Furthermore, the cartilage-targeting capacity could also be bestowed to the stimuli-responsive DDSs with controlled release capabilities and the ability to cross biological barriers and therefore enable releasing drugs precisely in response to internal or external stimuli, allowing them to enrich the site of damaged cartilage, thus producing enhanced therapeutic effects with minimal systemic side effects [31,32]. This paper describes various delivery systems such as nanoparticles, liposomes, hydrogels, and microspheres and discusses their application and efficacy as DDSs for the treatment of OA. Factors affecting the delivery effects are then discussed and reviewed in terms of both endogenous stimuli (reactive oxygen species, pH, enzymes, and temperature) and exogenous stimuli (near-infrared (NIR) rays, ultrasound, and magnetic fields) (Figure 2). Finally, the challenges and potential solutions for the clinical application of stimuli-responsive DDSs are summarized.

## 2. Drug Delivery Systems

Due to the poor permeability of drugs (especially small molecules) into the dense cartilage matrix, as well as rapid clearance through synovial capillaries and lymphatics, various intra-articular injection DDSs have been developed in order to prolong the bioavailability and circulation time of drugs, such as nanoparticles, liposomes, hydrogels, microspheres, etc., as carriers to deliver drugs [33,34]. The following briefly describes several common clinical delivery systems, such as nanoparticles, hydrogels, liposomes, and microspheres, and discusses their use and efficacy as DDSs for the treatment of OA.

### 2.1. Nanoparticles

Since the approval of nanoparticles (NPs) by the U.S. FDA for therapeutic use in 1989, NPs have been used to a great extent in tissue engineering due to their size manipulation, variation of chemical and physical properties (depending on the material composition and cross-linking modes), surface modification, and high surface-to-volume ratio [35]. NPs are submicron particles with a size of 1~100 nm, about 1000 times smaller than chondrocytes. The controlled size of NPs makes them feasible for direct intra-articular injection. NPs as carriers can delivery drugs to the cartilage surface or matrix to protect them from enzymatic degradation, improve their ability to penetrate the cartilage matrix, and modulate the pharmacokinetics of the drug, which facilitates the balancing of efficacy and toxicity of therapeutic compounds [36]. For instance, Wei et al. developed a platform for secreted phospholipase A2 (sPLA2)-responsive NP interventions for OA by incorporating a sPLA2 inhibitor (sPLA2i) into micellar phospholipid membranes. Micellar NPs loaded with sPLA2i (sPLA2i-NPs) can penetrate deep into the cartilage matrix and prolong the retention time of the joint space, which can greatly slow down the progression of cartilage degeneration, reduce synovial inflammation, prevent the formation of bony encumbrances, and relieve joint pain [37]. Liang et al. prepared a melatonin-loaded nano-delivery system (MT@PLGA-COLBP) by encapsulating melatonin (MT) in poly (lactic-co-glycolic acid) (PLGA) using the water-in-oil method and then attaching a type II collagen-targeting peptide to the surface to prepare the MT-loaded delivery system. MT@PLGA-COLBP could maintain stability for at least 21 days and remain in the joint cavity of mice, releasing melatonin for at least 14 days. MT improves cartilage matrix metabolism and delays the progression of OA by inhibiting the TLR2/4-MyD88-NFκB signaling pathway, scavenging reactive oxygen species (ROS), and inhibiting the activation of the innate immune system. In addition, MT@PLGA-COLBP can reach the inner cartilage, reduce the number of intra-articular injections, and improve the utilization of MT in vivo [38]. In addition, gene therapy has been widely studied as a promising therapeutic strategy in many diseases [39], and intra-articular injections of therapeutic nucleic acids have been reported to prove to be an effective treatment for OA. Therefore, Chen et al. proposed the construction of functionalized 5th-generation (G5) polyamidoamine (PAMAM) dendrimer (named G5-AHP) with arginine, phenylalanine, and histidine as an effective vector for microRNA-224-5p delivery, which can concentrate microRNA-224-5p into transfected NPs with a higher cellular uptake and transfection efficiency and can protect microRNA-224-5p from degradation by RNase while regulating synovial inflammation to balance the homeostasis of OA. By developing this efficient gene vector, it is the key to the success of OA gene therapy [40]. Gui et al. synthesized porous polymer NPs (SOD-NPs) loaded with superoxide dismutase (SOD). The NPs delivered SOD to the mouse knee joint and accumulated mainly in the synovial membrane, which prolonged the retention time of SOD in the mouse joints and possessed highly efficient antioxidant properties [41].

Cartilage consists of a highly anionic matrix, because its structure consists of negatively charged glycosaminoglycans (GAGs), which provide highly competent binding sites for positively charged drugs, which is advantageous for enhancing the absorption of arthritis drugs and prolonging the retention of drugs in the tissue [42]. Considering this factor, Kumar et al. designed cationic particles smaller than 20 nm to promote their retention in cartilage. NPs made of manganese dioxide (MnO_2_) for scavenging ROS were developed. MnO_2_ NPs were first designed to have physicochemical properties that promote cartilage resorption and the penetration of cartilage and to prolong their retention in the joint. By intra-articular injection, these particles demonstrated the ability to penetrate the cartilage extracellular matrix and co-localize with resident chondrocytes both in vitro in cartilage explants and in vivo. The longevity of the particles in the joints may also be related to their electrostatic interaction with the anionic cartilage matrix. Thus, MnO_2_ NPs in vivo not only prolong joint retention but also localize to cartilage [43]. Mg^2+^ is the fourth most common divalent cation in the human body and the second most abundant intracellular cation, being stored mainly in the skeletal system, where it plays a crucial role in maintaining bone and cartilage health. Zheng et al. developed PLGA microspheres loaded with nano-magnesium oxide (MgO) modified with stearic acid for intra-articular injection. By injecting PLGA microparticles containing MgO NPs in the joint cavity, Mg^2+^ could be released stably without changing the local acid–base environment. Furthermore, Mg^2+^ promoted chondrogenic differentiation and inhibited osteogenic differentiation of bone marrow mesenchymal stem cells (BMSCs). At the same time, it inhibited chondrocyte apoptosis and osteoblast formation through an AKT-related pathway, effectively mitigating the progression of OA, while cartilage and subchondral bone were simultaneously affected [44].

Collectively, although NPs can penetrate deep into the cartilage matrix due to their small size, which also brings problems needed to be avoided, such as endocytosis by immune cells. Therefore, NPs always need to be modified to enhance their joint retention time.

### 2.2. Hydrogels

**Figure 3:**
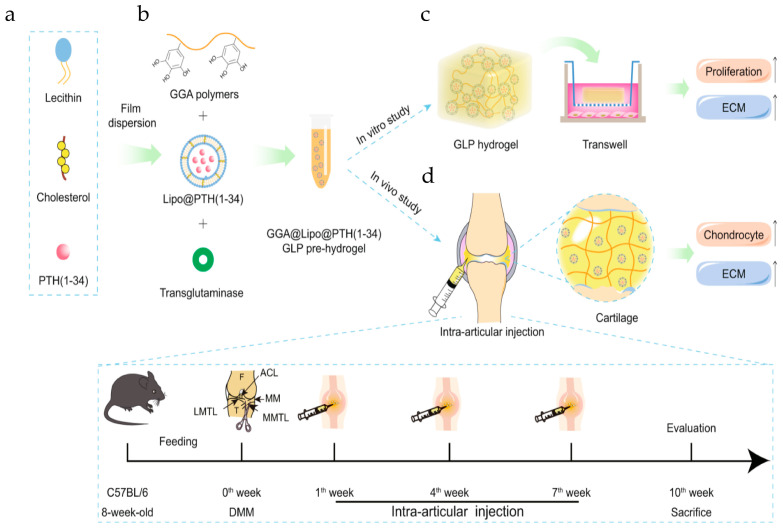
Schematic of the fabrication of an injectable hydrogel system and its main research ideas. (**a**) The preparation of liposomes loaded with PTH (1–34) (Lipo@PTH (1–34)) by means of the film dispersion method. (**b**) The preparation of GGA@Lipo@PTH (1–34) (GLP) hydrogel. (**c**) In vitro study for the GLP hydrogel, including physicochemical properties’ characterization and in vitro ATDC5 cells culture study. (**d**) In vivo intra-articular injection of the GLP hydrogel and the treatment protocol for the in vivo study. ECM: extracellular matrix; DMM: destabilization of the medial meniscus. Reprinted with permission from Ref. [20], copyright 2023, Li et al.

Hydrogels are hydrophilic polymers consisting of three-dimensional physically or chemically interconnected networks that, due to their thermodynamic adaptation to water, can absorb large amounts of water while maintaining their structure [45]. It is a very promising subset of biomaterials due to its inherent characteristics, such as good biocompatibility, high water content, minimal cytotoxicity, and biodegradability [23]. When a drug is loaded into an injectable hydrogel during administration, the hydrogel serves as a reservoir for the slow release of the drug while maintaining a high local concentration for an extended period of time [46]. Yan et al. used DNA supramolecular hydrogels as BMSC carriers to treat OA. The results of this study showed that, compared to the current clinical strategy, DNA supramolecular hydrogels can significantly improve the viability of mesenchymal stem cells (MSCs) during the delivery process, provide excellent anti-friction protection, three-dimensional support, and a three-dimensional microenvironment for cell proliferation, differentiation, and migration and ultimately enhance the therapeutic efficacy of MSCs. This study provides a promising and innovative clinical strategy and insights into regenerative medicine for the treatment of OA with bone marrow MSCs [47]. As illustrated in Figure 3, Li et al. introduced a lipid-anchored teriparatide doped into gallic acid-grafted gelatin-injected hydrogels (GLP hydrogels). Briefly, parathyroid hormone (PTH (1–34)) was encapsulated in the core of liposomes by membrane dispersion, and then, liposomes loaded with PTH (1–34) (Lipo@PTH (1–34)) were added to gallic acid-grafted gelatin (GGA) solution and then formed into an injectable hydrogel by the transglutaminase (TG) enzyme cross-linking method. Based on this study, (i) it was demonstrated that GLP hydrogels can be formed in situ by intra-articular injection in mice and do not affect knee motion; (ii) the sustained release of PTH (1–34) from GLP hydrogels could be achieved when injected into mice; (iii) validation that GLP hydrogels promote the proliferation of mouse embryonic tumor cells (ATDC5) cells and protect against IL-1β-induced ATDC5 cells’ further development, possibly through the PI3K/AKT signaling pathway; (iv) confirming that GLP hydrogels protect against cartilage degradation, mitigate OA progression, and promote GAG synthesis when injected intra-articularly in destabilization of the medial meniscus (DMM)-induced OA mice [20]. Hu et al. prepared ADH@SeNP hydrogels by one-step mixing of oxidized hyaluronic acid (OHA) solution with adipic dihydrazide-grafted HA (HA-ADH) solution and selenium NP (SeNP) suspension based on the mild Schiff base reaction between OHA and HA-ADH, as well as the dispersion of SeNPs in the gel network. By further doping the hydrogel with SeNPs, it exhibited minimal toxicity, mechanical properties, self-healing ability, and sustained drug release, and it promoted cartilage repair through the synergistic effect of scavenging ROS and inhibiting apoptosis [48]. However, hydrogels derived from natural biopolymers exhibit inadequate mechanical properties, are prone to enzymatic degradation, and face challenges regarding structural integrity while potentially eliciting an immune response, all of which perhaps constrain their broader application [49,50,51].

### 2.3. Liposomes

Liposomes have excellent targeting and biodegradability compared to hydrogels [52]. Liposomes are vesicular structures formed by phospholipid bilayers around aqueous chambers with diameters ranging from 50 to 5000 nm. They can encapsulate a variety of active ingredients, such as hydrophobic and hydrophilic drug molecules, nucleic acids, etc. and are widely used to improve drug co-residence times [53]. Its phospholipid shell wraps hydrophobic drugs, and the nucleus wraps hydrophilic drugs. As a drug delivery carrier, liposome has good biocompatibility, drug stability, slow release, and other features [54]. Yang et al. proposed a hydrophilic coating-modified nano-liposomal drug carrier (PMPC-Lipo) to improve drug retention in the joint cavity. Carbon chain octadecylamine (ODA)-modified 4-cyano-4-(thiobenzoylthio) pentanoic acid was used as a chain transfer agent, and 2-methacryloyloxyethylphosphorylcholine (MPC) was polymerized with the modified chain transfer agent. The polymer obtained was combined with lecithin and cholesterol to form a liposome (PMPC-Lipo), and the poly (MPC) acted as a hydrophilic coating. PMPC-Lipo was found to restore the lubrication of mechanically damaged cartilage (simulating OA conditions) to the level of healthy cartilage due to hydrated lubrication. It was also found that PMPC-Lipo avoided macrophage recognition due to the presence of poly (MPC), thereby evading phagocytosis and prolonging its retention time within the synovial joint. In addition, after the encapsulation of gallic acid (GA) by PMPC-Lipo, the obtained GA-PMPC-Lipo could effectively scavenge ROS and restore the imbalance of matrix secretion in inflammatory chondrocytes. Overall, the proposed GA-PMPC-Lipo may provide new ideas for OA treatment by providing long-term effective drug action and excellent lubrication properties [55]. Liposomes are often combined with microcarriers or/and hydrogels to prolong drug retention in the joints [53]. Recent studies have shown that composite carriers of liposomes and hydrogel microspheres can combine the advantages of different material forms, exhibit greater plasticity and flexibility than traditional single carriers, and are expected to be a new local DDS [54]. Liu et al. developed an injectable nanoliposome-encapsulated hydrogel with self-lubricating and friction-responsive properties for the treatment of meniscal tears, inspired by the composition and function of the meniscus. The hydrogel acted as a reservoir for the nanoliposomes, loading the liposomes with diclofenac sodium (DS) and kartogenin (KGN), which are used for anti-inflammatory purposes and for cartilage regeneration, to ensure continuous exposure of the nanoliposomes and formation of a hydrated layer to minimize friction during movement. In addition, the drugs contained in the nanoliposomes exhibited slow released properties, thereby reducing inflammation and promoting cartilage regeneration [56]. However, liposomes have some drawbacks, such as leakage of encapsulated drugs, physical instability, and rapid clearance of slips [57]. These shortcomings pose challenges for lipid-based DDSs.

### 2.4. Microspheres

**Figure 4:**
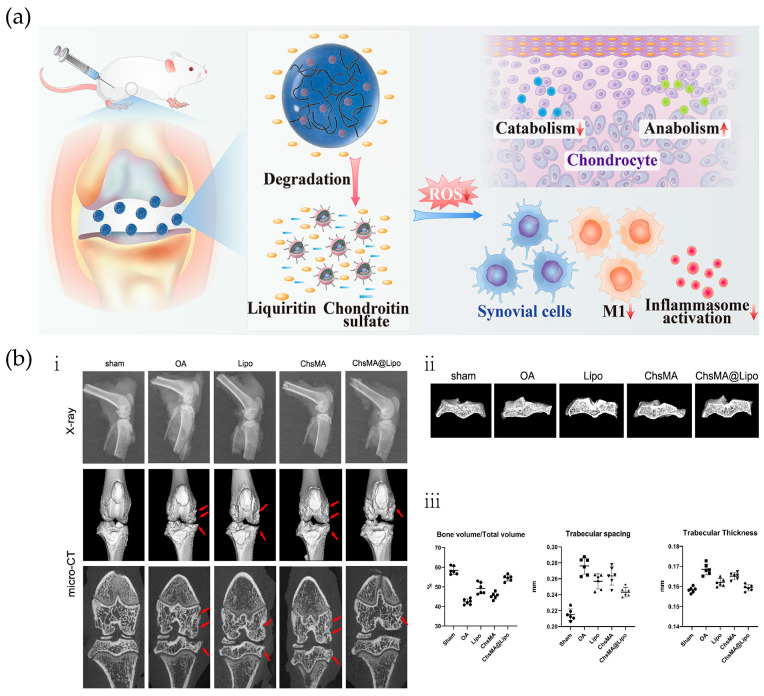
ChsMA@Lipo microspheres protect articular cartilage by eliminating ROS. (**a**) Effect of dual antioxidation of ChsMA@Lipo via intraarticular injection. LQ release overcomes double obstruction from the lipid membrane and the hydrogel matrix network. Microspheres can degrade into chondroitin sulfate through enzymatic action, which can synergize with LQ to eliminate ROS. (**b**) (**i**) Representative X-ray images and micro-CT images of knee joints in the different groups at 10 weeks post-surgery, showing the osteophyte differences in different groups (red arrow); (**ii**) changes in the subchondral bone in each treatment group; and (**iii**) quantitative analysis of the volume fraction (bone volume over total volume; BV/TV), trabecular spacing (T.p.), and trabecular thickness (T.h.) of the MTPs in the five groups 10 weeks post-surgery. Reprinted with permission from Ref. [54], copyright 2022, He et al.

Microspheres are small spherical entities formed by dissolving or dispersing drugs in polymeric materials, with particle sizes ranging from 1 to 250 μm. Microspheres are widely used because of their convenience in oral drug delivery, stable adsorption of drugs in microspheres, and less local irritation and adverse reactions caused by drug-loaded microspheres [58]. He et al. constructed injectable degradable microspheres with drug-carrying liposomes as secondary structures. Injectable chondroitin sulfate hydrogels were prepared by covalently modifying chondroitin sulfate with photo-cross-linked methacryloyl (ChsMA), and liquiritin (LQ)-loaded liposome-anchored ChsMA microgels (ChsMA@Lipo) were developed to delay the progression of OA through dual antioxidants (Figure 4a). With the degradation of microspheres in vivo, chondroitin sulfate can synergize with LQ to exert antioxidant effects to effectively eliminate ROS to protect articular cartilage and inhibit cartilage matrix degradation. The therapeutic effect can be assessed by radiographs and micro-CT scans of treated OA rats (Figure 4b). Thus, ChsMA@Lipo is a promising option for OA treatment as a degradable dual antioxidant drug delivery platform [54]. Yao et al. used sulfhydryl polyhedral oligomeric silsesquioxane (POSS-SH) as a nano-constructive platform to chemically graft polyethylene glycol (PEG), KGN, hydrogenated soybean phosphatidylcholine (HSPC), and fluorescein (PPKHF) in a one-step “click chemistry” approach. A stable, positively charged, multifunctional POSS hybrid molecule was formed, combining fluorescence imaging, the promotion of cartilage repair, and enhancement of joint lubrication. Then, PPKHF was homogeneously mixed with methacrylic acid hyaluronic acid to prepare lubricating hydrogel microspheres (MHS@PPKHF), which have a strong retention effect in the joint cavity, facilitating the visual monitoring of drug metabolism, which is conducive to clinically individualized treatment and observation [59]. Han et al., inspired by the hyper-lubricating properties of cartilage and the catecholamine chemistry of mussels, successfully developed injectable hydrogel microspheres with enhanced lubrication and controlled drug release for OA therapy. Specifically, GelMA microspheres were prepared using microfluidic technology and then impregnated and coated with DMA-MPC bionic lubrication coating, which was successfully developed in a previous study, to generate lubricated hydrogel microspheres (GelMA@DMA-MPC). Subsequently, GelMA@DMA-MPC microspheres were loaded with the anti-inflammatory drug DS and intra-articularly injected into the rat knee joint. A series of experiments demonstrated that GelMA@DMA-MPC microspheres prevented OA degeneration through the synergistic treatment of enhanced lubrication (friction coefficient reduction) and sustained drug release (inflammation downregulation) [60]. Combining simulated cartilage lubrication properties with sustained drug delivery has shown therapeutic potential in animal models and warrants further investigation in human clinical trials.

## 3. Controlled Stimulus Response DDSs

Despite the advancements in drug delivery systems, the current targeted DDSs exhibit several limitations, including inadequate flexibility, suboptimal nano-properties, unsatisfactory cycle durations, low targeting efficiency, poor tissue penetration, uncontrolled drug release profiles, challenges in ensuring patient compliance, and a requirement for higher dosages with more frequent administration [61,62]. The ideal DDSs would be able to cross biological barriers to release the desired concentration of active drug at the appropriate time and site of action. Stimuli-responsive drug delivery vehicles are emerging to ensure highly specific and sensitive drug delivery [63]. Stimulus-responsive materials are a new class of smart materials developed based on the concept of bionics, which can respond to small changes in the environment through significant changes in chemical or physical properties, such as temperature, pH, magnetic field, light, and ultrasound [64]. Thus, stimuli-responsive DDSs can take advantage of specific microenvironmental conditions or external stimuli at the lesion site to specifically release the active therapeutic ingredient at the desired site in a controlled and targeted manner [65]. Due to their functional properties, stimuli-responsive materials have a wide range of applications in the biomedical field. Once a stimuli-responsive DDS has been designed to load a drug into a carrier, the on-demand release of the drug can be ideally achieved depending on the different disease/physiological environments or the dynamic processes of the organism itself. Therefore, when designing a new drug carrier, the first consideration is the physiological environment in which the drug carrier enters the diseased cells or tissues of the body [66]. The responsive nano-delivery carriers are classified into two broad categories: endogenous stimulus responses (redox reactions, pH, enzymes, and temperature) and exogenous stimulus responses (NIR, magnetic, and ultrasound) based on the exogenous and in vivo environments, as well as the source of the stimulus responses, and their clinical applications in articular cartilage regeneration and repair are reviewed.

### 3.1. Endogenous Stimulus-Responsive Delivery Systems

Under the pathological conditions of OA, the balance between antioxidants and ROS is disrupted by the depletion of antioxidants, excessive accumulation of ROS in cartilage and synovium, or both. This imbalance in cellular redox results in oxidative stress and damage to chondrocytes, leading to cartilage degradation [41,67]. In addition, TNF-α and IL-1 produced by resident chondrocytes and synoviocytes are thought to be important cytokines that promote disease propagation. IL-1 signaling stimulates chondrocytes to produce MMPs (e.g., MMP-1, -3, and -13) and aggregating glycolytic enzymes (e.g., integrins with platelet-reactive protein motifs and metalloproteinases; ADAMTS-4 and -5) [68]. IL-1 also inhibits proteoglycan and type II collagen synthesis while promoting an increased production of reactive oxygen factors, such as nitric oxide [69,70]. Considering the irritants in OA joint tissues, the development of stimuli-responsive DDSs with controlled loading of anti-inflammatory drugs inhibit inflammatory factor-induced cell destruction, thereby slowing down the progression of OA. The advanced applications of endogenous stimuli (redox system, pH, enzymes, and temperature) nanomaterials in OA therapy are reviewed below (Table 1).

#### 3.1.1. Reactive Oxygen Response

**Figure 5:**
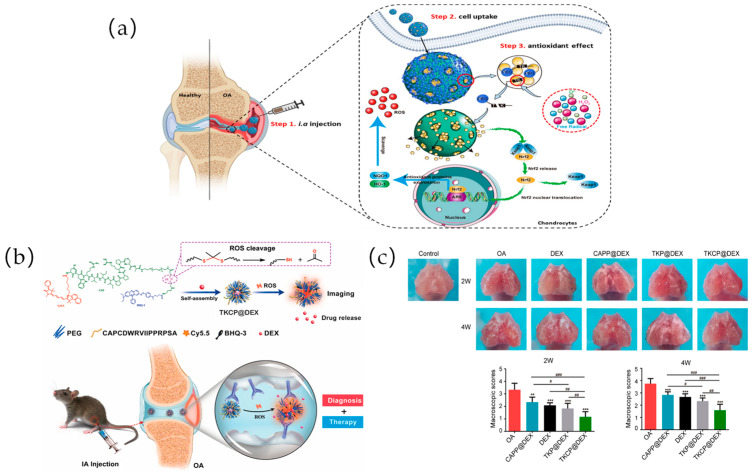
ROS-responsive biomaterials for OA treatment: (**a**) Inspired by oxidative stress in the pathogenesis of OA, a novel antioxidant, OL, was loaded on the mesoporous silica NP, which was modified with methoxy polyethylene glycol-thioketal to gain the ROS-responsive ability. MSN-OL could be easily up taken by chondrocytes and releases OL when the cells are injured and in a state of hyper-oxidation. MSN-OL can further activate the Nrf2/HO-1 signaling pathway and exhibit antioxidant and antiapoptotic ability. Reprinted with permission from Ref. [72], copyright 2022, Jiang et al. (**b**) Schematic of the self-assembly of ROS-responsive NPs for in vivo bioimaging and targeted therapy of OA. Reprinted with permission from Ref. [75], copyright 2021, Shen et al. (**c**) Macroscopic appearance and scoring of cartilage femoral condyles after treatment for 2 and 4 weeks. The macroscopic observation and the macroscopic scores of cartilages after IA injection with PBS, DEX, CAPP@DEX, TKP@DEX, or TKCP@DEX (n = 6; ean  ±  SD; # indicate *p*  <  0.05, **; ## indicate *p* <  0.01; ***, ### indicate *p*  <  0.001). Reprinted with permission from Ref. [75], copyright 2021, Shen et al.

ROS are highly reactive ions and free radicals, including superoxide (O_2_^−^), hydroxyl radical (·OH), hypochlorite ion (OCl^−^), hydrogen peroxide (H_2_O_2_), and monoclinic oxygen (^1^O_2_) [34,88]. ROS play a crucial role in physiological functions, including the regulation of protein function, production of multiple hormones, modulation of cell signaling, mediation of inflammation, and elimination of pathogens. In general, low levels of ROS regulate cell signaling pathways and promote cell proliferation [89]. However, excessive ROS can disrupt normal signal transduction and homeostasis [90]. Based on the elevated levels of ROS specific to certain diseases, polymeric nanocarriers with distinct ROS-responsive properties may enhance targeted drug delivery for the treatment of these conditions [91]. To address the problem that the dense extracellular matrix of cartilage is poorly targeted and rapidly cleared from the cartilage surface, which prevents NP penetration and requires repetitive high-dose administration within the joint cavity, Wu et al. proposed the use of the ROS-responsive material poly(ethylene glycol diacrylate) (PEGDA)-1,2-ethylenedithiol (EDT) copolymer (PEGDA-EDT) and reduced graphene oxide (rGO) as graphene-based nanomaterials to prepare a smart ROS-responsive poly (lactic acid) (PLA)/PEGDA-EDT@rGO-fucoxanthin (PPGF) nanofibrous membrane as a DDS for OA therapy. PPGF nanofibrous membranes were prepared by introducing PEGDA-EDT as a ROS-responsive motif, rGO as a drug carrier, and fucoxanthin (Fx) as an antioxidant and anti-inflammatory agent in PLA to achieve ROS-responsiveness and long-term drug release for OA therapy. The novel nanofibrous membrane DDS could “turn on” in response to excessive ROS, prolonging the residence time of Fx in the joint cavity, promoting effective drug concentration, and improving the therapeutic efficiency of OA [71]. Inspired by oxidative stress in the pathogenesis of OA, Jiang et al. first demonstrated the antioxidant capacity of the small molecule compound oltipraz (OL) on IL-1β-treated chondrocytes. Then, a functional ROS-responsive nanocarrier, i.e., mesoporous silica NPs (MSNs) modified with methoxy polyethylene glycol-thioketal (TK) and loaded with OL, was constructed (Figure 5a). In this study, by constructing a new type of nanocarrier, the antioxidant can be brought into chondrocytes, and the drug can be intelligently released in a high-ROS environment [72]. Zhang et al. reported an innovative ROS-responsive therapeutic polymer NP capable of loading hydrophobic drugs and self-reporting the payload release upon ROS stimulation. The NPs consist of an amphiphilic block copolymer composed of PEG and an oxidatively responsive hydrophobic block containing pendant phenylboronic pinacol ester groups and a small portion of a 1,8-naphthimide dye. Since the naphthimide dye is covalently coupled to the polymer, it is possible to fluorescently track the polymer carrier inside the cell [73]. Wu et al. synthesized and assembled a hydrogen peroxide (H_2_O_2_, which belongs to the ROS)-sensitive nanomicelle loaded with the anti-inflammatory drug dexamethasone (DEX) and the cartilage differentiation factor chondrocyte-derived photoproduced protein-1 (CDMP-1). This low-toxicity, ROS-responsive NP (DLNP) capable of eliminating joint inflammation and inducing cartilage repair was constructed. H_2_O_2_ was used as a positive control, and the NPs had -SeSe- groups as the ROS-responsive component, with DEX and CDMP-1 as the main pharmacophore. The drug-carrying NPs were delivered directly to the arthritic lesions by intra-articular injection. Using the high concentration of oxygen radicals at the arthritic lesion, -SeSe- breakage and slow release of DEX and CDMP-1 were observed. The results showed that the drug-loaded micelles effectively inhibited the proliferation of activated macrophages, induced macrophage apoptosis, and had anti-inflammatory effects and led to the differentiation of BMSCs to chondrocytes [74]. Shen et al. developed a multifunctional ROS-activated therapeutic polymer NP capable of loading hydrophobic drugs and self-reporting the payload release upon ROS stimulation. The NPs consisted of a Cy5.5-modified cartilage-targeting peptide (CAP, DWRVIIPPRPSA) and a PEG-modified oxidative-responsive TK ligand hydrophobic block containing black hole quencher 3 (BHQ-3) as a quencher for Cy5.5, which was then encapsulated with DEX to form the TKCP@DEX NPs. TKCP@DEX NPs specifically targeted articular cartilage via CAP and responded to high levels of ROS due to high levels of ROS in inflamed tissues leading to sulfhydryl bond breakage, resulting in the gradual breakdown of the polymer and release of Cy5.5 and the drug (Figure 5b). The results show that the nanoprobes can be intelligently “switched on” in response to excessive ROS and “switched off” in normal joints. Especially for cartilage, TKCP@DEX can effectively respond to ROS and slow-release DEX to significantly reduce cartilage damage in OA joints. An in vivo experiment was conducted to evaluate the therapeutic effect of TKCP@DEX on OA. By observing the macroscopic appearance and scores of the cartilaginous femoral condyles at 2 and 4 weeks after treatment, the results showed that the TKCP@DEX group reduced the damage by 65% and 57.78%, respectively, which demonstrated its effective restorative effect in the treatment of OA (Figure 5c) [75]. Tang et al. reported an innovative self-reporting drug delivery platform based on ROS-responsive random copolymers (P1) capable of visualizing drug release kinetics through activation of an integrated fluorophore. P1 is synthesized by co-polymerization of boronic acid pinacol, PEG, and naphthalene dicarbonyl imide monomers, which endow ROS sensitivity, hydrophilicity, and fluorescence signals, respectively [76]. ROS-responsive materials have been the focus of numerous studies as one of the triggering drug release mechanisms for responsive polymeric DDSs. ROS are overexpressed at high concentrations at many disease sites. The differences between normal and pathological tissues make ROS-responsive delivery systems with great potential for many disease applications [92].

#### 3.1.2. pH Response

Due to the presence of an acidic microenvironment in a variety of pathophysiological conditions, pH-responsive materials have been extensively investigated for the site-specific delivery of different therapeutic agents in formulations across multiple scales [93]. For example, in diseased tissues such as cancer, bacterial infections, and inflammation, the pH is different from the physiological pH 7.4, and therefore, pH-responsive DDSs are becoming increasingly popular, which can take advantage of this pH difference to specifically release encapsulated drugs into diseased tissues [58]. Thus, by designing nanomaterials to release drugs at a specific pH, drugs can be selectively delivered to the site of inflammation and released to produce a therapeutic effect, and one way to design pH-responsive polymeric nanomaterials is to use polymers with pH-sensitive functional groups. These functional groups can change conformation or decompose in response to changes in pH, resulting in the release of the drug from the nanomaterials [94]. There are two mechanisms for the release of pH-sensitive drug carriers: the first stems from changes in hydrophobicity or charge of the carrier induced by protonation or deprotonation due to changes in the ambient pH, and the other is caused by dynamic chemical bond breaking in pH-sensitive biomaterials [95]. Xiong et al. developed a pH-responsive metal–organic frameworks (MOFs) system modified by HA and loaded with the anti-inflammatory protocatechuic acid (PCA), named MOF@HA@PCA, for the treatment of OA. The results showed that MOF@HA@PCA was able to respond intelligently to the acidic conditions in the OA microenvironment and gradually release the PCA, thereby significantly reducing IL-1β-induced synovial inflammation in chondrocytes and OA joints. MOF@HA@PCA also downregulated the expression of OA inflammatory markers and promoted the expression of cartilage-specific genes [77]. He et al. designed a nanoscale pH-responsive DDS for OA therapeutics by using modified MSNs and pH-responsive poly (acrylic acid) (PAA) loaded with andrographolide (AG) to form AG@MSNs-PAA nanoplatforms. The NPs have uniform size (∼120 nm), high drug loading efficiency (22.38 ± 0.71%), and pH-responsive properties, which are beneficial for slow release in OA environments [78].

Zhang et al. designed a super-lubricating circulating brush amphoteric polymer (CB). Specifically, the cyclic polymer poly-2-hydroxyethyl methacrylate (c-p(HEMA)) was used as a core template for the statistical copolymerization of [2-(methacryloyloxy) ethyl]dimethyl-(3-sulfopropyl) (SBMA) and *N*,*N*-dimethylaminoethyl methacrylate (DMAEMA) by “one-step” atom transfer radical polymerization to prepare pH-responsive cyclic electrically brushed amphiphilic polymers (cb-P(HEMA-g-P(DMAEMA-st-SBMA)), CB). In particular, the special structure of the ring–brush polymer can encapsulate both the anti-inflammatory hydrophobic agent curcumin (Cur) and the hydrophilic agent loxoprofen (LXP) to achieve a high drug loading capacity and then achieve a fast release of LXP and a slow release of Cur based on the pH-responsive behavior for long-term OA therapy [79]. In the case of OA, there is a decrease in synovial fluid pH in different regions of the synovial cavity, and in order to take advantage of these pH differences. Zerrillo et al. prepared PLGA NPs and ammonium bicarbonate (NH_4_HCO_3_)-encapsulated pH-responsive PLGA NPs. These NPs were loaded with HA as a possible model drug for OA and with NIR dyes for use in molecular imaging through the visualization of the NPs. This is because the porous surface of PLGA NPs allows small molecules such as H_2_O and H_3_O^+^ to enter. Under low pH conditions, high concentrations of hydrated hydrogen ions (H_3_O^+^) react with NH_4_HCO_3_ loaded in the NPs to produce NH_4_^+^, CO_2_, and H_2_O, leading to pH neutralization. Furthermore, it was shown that pH-responsive NPs exhibited extracellular burst release behavior and higher chondrocyte viability [80]. In addition, a pH-responsive NP drug with high delivery efficiency was constructed by loading celastrol into hollow HMSNs using chitosan as a coating, which also showed potential against OA [81].

#### 3.1.3. Enzyme Response

Enzymes play an important role as catalysts in a variety of biochemical reactions, and certain enzymes are upregulated under pathological conditions. These enzymes overexpressed in disease states have attracted scientists to develop enzyme degradable DDSs. In addition, enzyme-responsive materials are highly specific and selective with fast response rates compared to other stimulus-sensitive systems [96]. Since enzymes are highly selective and efficient, a variety of modes of drug release behavior, such as surface ligand activation and chemical bond breaking, can be achieved by being introduced into biomaterials functional groups that are sensitive to enzymes that are aberrantly expressed in the OA microenvironment [97]. Joshi et al. developed an arthritic flare-responsive hydrogel platform by self-assembling small molecule amphiphilic triglyceride monostearate (TG-18). The corticosteroid triamcinolone acetonide (TA) was loaded onto the TG-18 hydrogel as a model drug, and the drug reservoir released the drug in response to enzymes overexpressed in inflamed joints [82]. Yi et al. developed injectable hydrogels with MMP-responsive and sustained dexamethasone sodium phosphate (DSP)-releasing behavior for OA treatment. Oxidized hyaluronic acid (OHA) was prepared by the specific oxidation of HA by sodium periodate, and then, Col-OHA hydrogels loaded with DSP were prepared based on the Schiff base reaction between type I collagen and OHA, as well as the self-assembly of collagen. In addition, Col-OHA hydrogels showed a sustained release of MMP-responsive DSP, which had the ability to provide the sustained and effective relief of OA symptoms in vivo and had no significant effect on liver and kidney functions [83].

#### 3.1.4. Temperature Response

**Figure 6:**
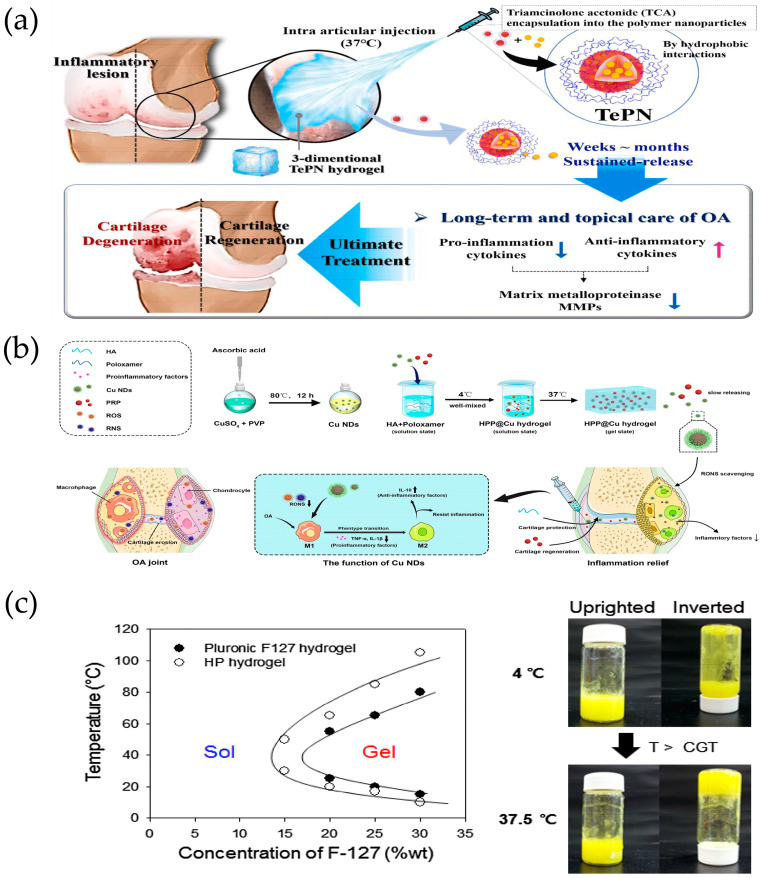
Temperature-responsive biomaterials for OA treatment: (**a**) Schematic diagram of a one-time injection of the TePN hydrogel system for the long-term treatment of OA. Reprinted with permission from Ref. [84], copyright 2022, Seo et al. (**b**) Schematic illustration for the synthesis processes of HPP@Cu thermos-responsive hydrogel and treatment mechanism as an articular microenvironment purifier for OA. Reprinted with permission from Ref. [85], copyright 2022, Zhu et al. (**c**) Sol–gel transition curves of Pluronic F-127 (●) and HP hydrogel (○). Photographic images of the PX-loaded HP hydrogel obtained at 4 and 37.5 °C. Reprinted with permission from Ref. [87], copyright 2017, Jung et al.

Thermosensitive hydrogels are designed based on the temperature differences between the human body and the external environment, as well as the variations among different tissues and organs [21]. When the temperature rises, the polymer chains dehydrate, facilitating the formation of hydrophobic domains. Driven by the increase in entropy, this process ultimately converts the hydrated liquid into a hydrogel network. This hydrogel can rapidly gel at cartilage defects during the transition from room temperature to physiological temperature (37 °C) [23]. Due to the interactions between its hydrophilic and hydrophobic domains, temperature-responsive in situ gels have garnered significant attention in tissue engineering. Seo et al. synthesized amphiphilic poly(organophosphazene) with temperature-dependent NP formation and sol–gel transition behavior when dissolved in aqueous solution for tretinoin (TCA) delivery. Since the hydrophobic portion of the polymer could interact with the hydrophobic portion of TCA, TCA was encapsulated into self-assembled polymer NPs (TePNs) that were well dispersed in aqueous solution at room temperature. The TePN solution can be injected in a solution state at around room temperature and converts directly to a hydrogel upon intra-articular injection due to body temperature. The encapsulated TCA is then slowly released by loosening the TePN hydrogel network and degradation of the polymer. Finally, the prolonged release of TCA treats OA by inhibiting MMP expression, decreasing pro-inflammatory cytokine expression, and increasing anti-inflammatory cytokine expression within the cartilage (Figure 6a) [84].

Zhu et al. developed a multifunctional composite thermosensitive hydrogel (HPP@Cu gel) consisting of a mixture of poloxamer 407 (P407) and HA used as a gel matrix, which was then physically mixed with copper nanodots (Cu NDs) and platelet-rich plasma (PRP). The HPP@Cu hydrogel was injected into the joint as a solution, which undergoes a phase change to a hydrogel at body temperature, slowly releasing the Cu NDs, HA, and PRP and prolonging the duration of pharmacological effects (Figure 6b) [85]. Li et al. designed a flurbiprofen thermosensitive gel based on poly(ε-caprolactone-co-lactide)-b-poly(ethylene glycol)-b-poly(ε-caprolactone-co-lactide) (PCLA-PEG-PCLA) triple-block copolymers for continuous intra-articular drug delivery. The copolymer system presents a flowable sol at room temperature to achieve its injectability and rapidly converts to a stationary gel at body temperature to form a drug reservoir that serves as a slow-release matrix for intra-articular drug delivery. In vitro drug release studies have demonstrated the sustained release of flurbiprofen from thermosensitive gels for more than three weeks. The intra-articular administration of flurbiprofen was effective in prolonging analgesia and significantly reduced the inflammatory response in a rat model of OA by downregulating the expression of inflammatory factors [86]. Jung et al. showed a novel intra-articular injectable thermosensitive hydrogel for long-term delivery of the nonsteroidal anti-inflammatory drug piroxicam (PX) that was prepared by a simple physical mixing of HA and Pluronic F-127 (HP) in aqueous solution. Pluronic F-127, as a biocompatible polymer, exhibits a rapid thermos-reversible sol–gel transition behavior and the ability to stabilize hydrophobic drugs in aqueous solution (Figure 6c), and biocompatible polysaccharide HA was used as a carbohydrate additive for controlled drug release to reduce the concentration of Pluronic F-127 required for gelation. Both in vitro and in vivo results showed that PX-loaded HP hydrogels not only exhibited excellent mechanical strength but also induced sustained drug release behavior [87].

### 3.2. Exogenous Stimulus-Responsive Delivery Systems

The study of endogenous stimuli is elusive, and intrinsic stimuli are relatively complex and uncontrollable [98]. External stimuli can remotely regulate the activation of nanomaterials through exogenous means. Stimulation can be manually controlled and adjusted during treatment to accommodate individual requirements [99,100]. Indeed, external physical stimuli, such as magnetic or electric fields, acoustic waves, and electromagnetic radiation, can not only be specifically positioned on the target to gain spatial control over drug release but also allow easy control of the exposure time and the intensity of the applied stimulus [99]. A review of advanced applications of exogenously stimulated nanomaterials in the treatment of OA in terms of NIR therapy, magnetism, and ultrasound is presented below (Table 2).

#### 3.2.1. NIR Response

**Figure 7:**
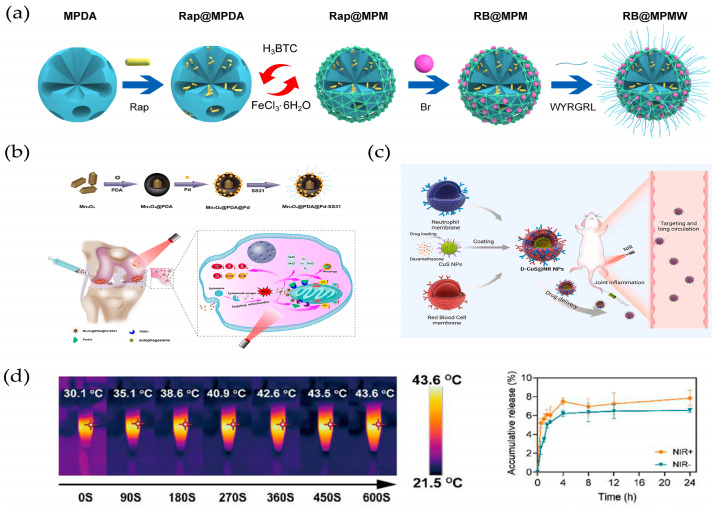
NIR-responsive biomaterials for OA treatment: (**a**) The mechanism of a dual-drug delivery nanoplatform with a cartilage-targeting effect and NIR laser response for OA therapy. Reprinted with permission from Ref. [101], copyright 2021, Xue et al. (**b**) Procedure schematic of Mn_3_O_4_@PDA@Pd-SS31 synthesis, and schematic illustration of Mn_3_O_4_@PDA@Pd-SS31, which performs to sequentially scavenge ROS and initiate mitophagy for treating OA. Reprinted with permission from Ref. [104], copyright 2023, Li et al. (**c**) Schematic diagram of the preparation and therapy of the D-CuS@NR NPs. Reprinted with permission from Ref. [105], copyright 2022, Xue et al. (**d**) Thermal images of CuS@NR NPs under 1064 nm NIR irradiation at 0.9 W/cm^2^ for 600 s (50 μg/mL), and drug release curves of D-CuS@NR NPs with or without a 1064 nm NIR laser in PBS medium (pH = 7.4) (n = 3). Reprinted with permission from Ref. [105], copyright 2022, Xue et al.

Due to the presence of photosensitive groups, photo-responsive hydrogels can respond to light with a specific wavelength (NIR, UV, or visible) under phase change. Remote, non-contact, and precise control of photo-responsive hydrogels can be achieved by light irradiation [21]. Photo-responsive DDS is a nano-DDS that enables drug release through photo-responsive materials under light irradiation at specific wavelengths. Compared to other internal stimulation methods, photo-stimulation has many advantages such as convenient operation, easy accessibility, and the ability to achieve precise control in time and space. In addition, external light stimulation can avoid individual differences caused by patients themselves [110]. Xue et al. prepared microspheres of mesoporous polydopamine (MPDA) wrapped in a metal–organic skeleton (MOF) and modified type II collagen-targeting peptide (WYRGRL) on their surface. A novel nanodrug-carrying system for OA treatment was obtained by loading rapamycin in the core layer of MPDA and bilirubin in the shell layer of MOF to realize the delivery of dual drugs (Figure 7a). The experimental results showed that the dual-DDS had excellent NIR laser-stimulated responsive drug release, which could sequentially achieve Br scavenging of cellular free radicals and enhancement of autophagic activity via Rap. Briefly, the rapid release of Br from MOF shells exhibited excellent ROS scavenging ability and antiapoptotic effects but responsively reduced autophagic activity to some extent. Meanwhile, Rap was rapidly released from the MPDA core under NIR irradiation, further enhancing autophagy activation and chondrocyte protection [101], since molybdenum-based polyoxometalate clusters (POMs), often described as nanoclusters of transition metals and oxygen atoms, have been shown to be promising candidates for ROS-related diseases [111].

In addition, several studies have shown that POM has excellent NIR response properties [112]. Shi et al. developed a NIR-responsive POM to treat an OA mouse model primarily by enhancing ROS scavenging. The results showed that NIR-responsive POM exhibited excellent antioxidant activity and anti-inflammatory effects, which could effectively alleviate the clinical symptoms of OA mice. Meanwhile, the progression of OA was slowed down by reducing the production of inflammatory cytokines and catabolic proteases [102]. He et al. developed a photothermal therapy injectable hydrogel for OA treatment, which was achieved by binding HA-thiourea (NCSN) solution to Cu^2+^ in a single-step process. The cross-linked structure of the hydrogel is based on the dynamically reversible chelation between NCSN and Cu^2+^. Notably, these cross-linking sites have significant photothermal properties. In addition, unchelated NCSN helps to neutralize excess ROS. When the hydrogel was used in combination with NIR therapy (HSC/NIR), it effectively promoted chondrocyte anabolism while reducing IL-1β-induced catabolism and inflammatory responses [103]. Li et al. innovatively constructed mitochondria-targeted and SOD-mimicking Mn_3_O_4_@PDA@Pd-SS31 nanoenzymes with good biocompatibility, NIR responsiveness, and a synergistic cascade for the scavenging of mROS for the treatment of OA. Briefly, Mn_3_O_4_ was used as the core of the nanoenzymes, encapsulated with PDA to enhance its biocompatibility and loaded with Pd to synergistically enhance the enzyme catalytic activity of the nanoenzymes. In addition, the mROS were precisely scavenged by modifying the mitochondria-targeting peptide SS31 on its surface to achieve a mitochondria-targeting ability. The results showed that the nano-enzymes accelerated the release of Pd and Mn_3_O_4_, enhanced the activities of SOD and CAT mimetic enzymes, reversed mitochondrial dysfunction, promoted mitochondrial autophagy, effectively scavenged mROS in chondrocytes, regulated the microenvironment of oxidative stress, and ultimately inhibited inflammatory responses under NIR irradiation (Figure 7b) [104]. Xue et al. prepared neutrophil and erythrocyte hybrid membrane-encapsulated dexamethasone sodium phosphate (Dexp)-loaded hollow copper sulfide NPs (D-CuS@NR NPs) for OA treatment (Figure 7c). Overall, synergistic treatment with mild heating, extended circulation, and targeted delivery was achieved in this system. The biomimetic NPs exhibited excellent photothermal conversion and controlled drug release behavior under a 1064 nm NIR laser (Figure 7d). Notably, D-CuS@NR NPs combined with 1064 nm NIR treatment had stronger anti-inflammatory effects at the cellular level compared to free Dexp and D-CuS@NR NPs. Meanwhile, in vivo fluorescence imaging showed that D-CuS@NR NPs could target inflammatory joint sites due to the activation of CD11a-containing neutrophil membranes. Importantly, D-CuS@NR NPs treated with NIR could effectively inhibit cartilage degeneration through photothermal therapy and the downregulation of synovial inflammation [105].

#### 3.2.2. Magnetic Response

Magnetic particles are nanostructures in the size range of 1–100 nm, usually containing a central magnetic core and a surface coating adjacent to a functional coating. Magnetic particles are generally composed of pure metals such as iron, cobalt, nickel, manganese, etc., and the main ones used for biomedical applications are iron oxide NPs [113]. Furthermore, magnetic particles can be used to overcome the problems of conventional DDSs and deliver drugs to the desired target area, maintaining the NPs in a specific location during drug release when combined with an external magnetic field [114]. Jiang et al. constructed a magnetically guided biodegradable nanocarrier system (MNP) and synthesized KGN-loaded magnetic NPs (KGN-MNPs) by encapsulating KGN on the surface of iron oxide NPs using poly(propylene lactone) (PLA). This system enabled intrachondral delivery of KGN to promote chondrogenic differentiation of bone marrow MSCs. In addition, the application of MNPs and an external magnetic field significantly increased the retention of KGN in the joint matrix, facilitated the penetration of more KGN-MNPs into the cartilage, avoided the rapid clearance of KGN from the joint, and improved the utilization of KGN [106].

**Figure 8:**
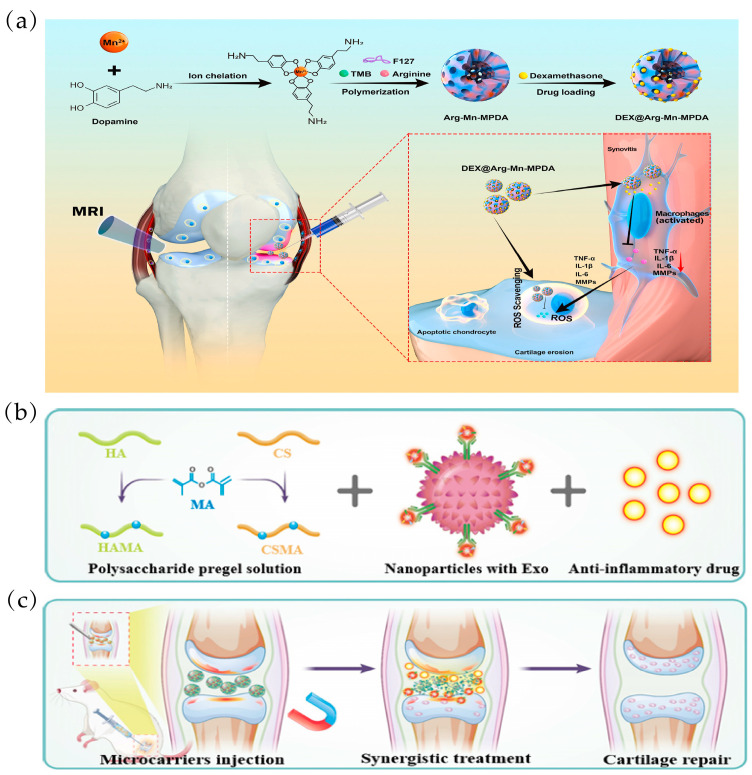
Magnetic-responsive biomaterials for OA treatment: (**a**) Schematic representation of DAMM NPs for the treatment of OA by anti-inflammatories and antioxidants. Reprinted with permission from Ref. [107], copyright 2023, Liu et al. (**b**) HA/CS-based polysaccharide pre-gel solution containing anti-inflammatory drug DS and magnetic Fe_3_O_4_@MgSiO_3_ NPs with captured Exo. Reprinted with permission from Ref. [108], copyright 2024, Yang et al. (**c**) Effects of MPM on OA treatment. Reprinted with permission from Ref. [108], copyright 2024, Yang et al.

Liu et al. developed mesoporous polydopamine NPs (DAMM NPs) doped with arginine and manganese ions (Mn) to deliver DEX for OA treatment (Figure 8a). Firstly, the mesoporous structure may enable AMM with a high specific surface area and abundant pores to load DEX more efficiently and prolong its release to inhibit synovial macrophage-induced inflammation and directly ameliorate OA progression. Secondly, arginine-doped PDA NPs can directly reduce ROS-induced chondrocyte apoptosis. In addition to these therapeutic properties, the incorporation of Mn^2+^ into arginine-doped MPDA NPs displays magnetic resonance imaging (MRI)-sensitive signals, enabling real-time visualization of damaged articular cartilage [107]. Yang et al. developed a magnetic polysaccharide hydrogel microsphere (MPM) consisting of modified natural polysaccharide hyaluronic acid (HAMA) and chondroitin sulphate (CSMA), which was prepared using microfluidic electrospray and freezing processes. Magnetic NPs with a spine-like structure capable of trapping stem cell exosomes (Exo) were encapsulated in a microcarrier, along with the anti-inflammatory drug DS (Figure 8b). The presence of magnetic NPs gives the microcarriers magnetic control (Figure 8c). In addition, DS and Exo released from the microcarriers had a synergistic effect on relieving OA symptoms and promoting cartilage repair. The results of in vitro and in vivo experiments demonstrated the excellence of microcarriers in OA treatment [108].

#### 3.2.3. Ultrasonic Response

Ultrasound is the “remote control” and “trigger” of smart composite biomaterials. It is mainly based on cavitation, microfluidics, scattering, and acoustic radiation force and other basic technical features to play a role in realizing the precise regulation of ultrasound-responsive biological components in smart composite biomaterials, such as driving, sensing, payload transfer, chemical or biological process initiation, etc. [115]. Ultrasound technology as a non-invasive method of precision medicine in the range of 0 kHz–50 MHz in combination with biomaterials has attracted great attention and has been widely used in the medical field [116]. Due to its non-invasiveness, ease of control, and high spatial and temporal precision, ultrasound can deliver drugs to blood vessels, tumors, and bone tissue, among others. Currently, ultrasound-triggered DDSs include micelles, liposomes, and hydrogels [117]. Jahanbekam et al. designed an injectable thermionic triggered drug carrier based on Pluronic^®^ F-127, HA, and gelatin. The optimized hydrogel was used in combination with four different surfactants, which could act as a reservoir for the drug to be released in a controlled manner to better regulate the release rate and provide a burst release when triggered by ultrasound [45].

Vinikoor et al. presented an injectable, biodegradable piezoelectric hydrogel made of short electrostatically spun poly-L-lactic acid nanofibers embedded within a collagen matrix, which can be injected into joints. In vitro experimental data showed that the piezoelectric hydrogel under ultrasound enhanced cell migration, induced stem cells to secrete TGF-β1, and promoted cartilage formation. In vivo, rabbits with osteochondral critical size defects receiving ultrasound-activated piezoelectric hydrogels showed increased subchondral bone formation, improved hyaline cartilage structure, and good mechanical properties close to healthy natural cartilage [118]. Yuan et al. reported PLGA MPs loaded with KGN (MPs@KGN) prepared by the premixed membrane emulsification (PME) method, and ultrasound transducer was utilized to sonicate them. In addition, carboxymethyl chitosan oxidized CS (CMC-OCS) hydrogels were prepared by embedding MPs by the Schiff base reaction to prepare CMC-OCS/MP scaffolds. The cumulative release of KGN from the MPs exhibited a slow rate, and after ultrasound treatment, the MPs appeared to be significantly collapsed, allowing KGN to maintain a continuous concentration for at least 28 days [109].

## 4. Multi-Stimulus Response

**Figure 9:**
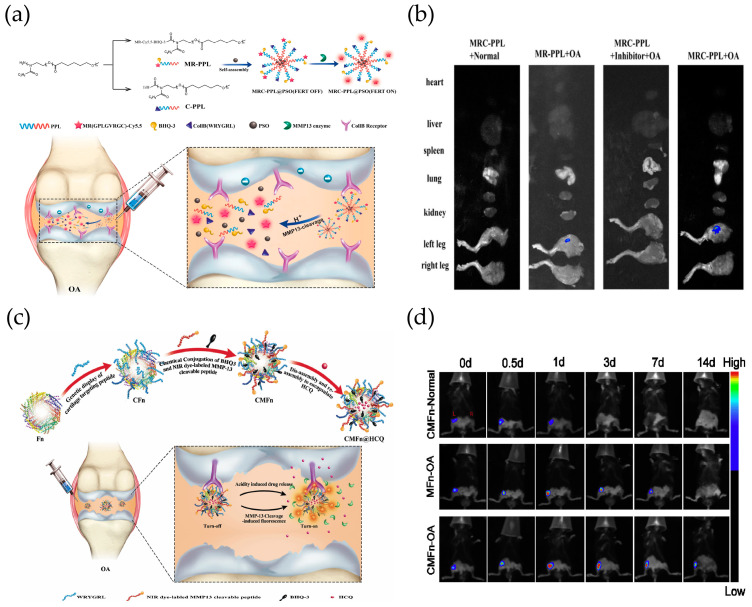
Multi-stimulus-responsive biomaterials for OA treatment: (**a**) Synthesis of MMP-13 and pH-responsive MRC-PPL@PSO nanomicelles, and schematic working mechanism for the treatment of OA. Reprinted with permission from Ref. [121], copyright 2020, Lan et al. (**b**) Ex vivo fluorescence imaging of the heart, liver, spleen, lung, kidney, left knee, and right knee at day 21 post-injection. Reprinted with permission from Ref. [121], copyright 2020, Lan et al. (n = 5, mean ± S.D.). (**c**) Schematic representation of CMFn@HCQ as a cartilage-targeted and MMP-13/pH dual-stimulation-activated therapeutic nanoprobe for in vivo MMP-13 imaging and precision therapy in OA. Reprinted with permission from Ref. [123], copyright 2019, Chen et al. (**d**) The fluorescence signal is measured by the IVIS Lumina II in vivo imaging system at a selected time point after IA injection. Reprinted with permission from Ref. [123], copyright 2019, Chen et al.

However, the development of OA is often accompanied by overall changes in the microenvironment of the joint cavity, such as an increased release of protein hydrolases induced by IL-1β and TNF-α, increased production of reactive oxygenating factors, such as nitric oxide, and a lower pH in the joint cavity compared to the normal joint cavity. Single stimuli-responsive drug delivery platforms no longer achieve well-targeted treatment of OA. For example, endogenous stimuli-responsive nanocarriers can ignore tissue permeation limitations but are challenging to administer in vitro. In contrast, exogenous stimulus-responsive nanocarriers are often limited by tissue penetration. Similarly, for many of the triggers listed above, the stimulus may not be specific for OA and result in unintended release of the drug [28]. Interestingly, the invention of multi-stimulus/multifunctional response nanocarriers through the rational design of multiple stimulus-sensitive moieties integrated on a single carrier realized the advantages of the synergistic action of multiple systems in a single system, amplified the carrier performance, and realized the precise concentration of the response in “real time” and the self-control of the drug release at a specific site [63]. Therefore, multi-stimuli-responsive nanotechnology drug delivery platforms have been reported clinically for the treatment of diseases, such as pH/redox-responsive, MMP/pH-responsive, pH/thermal-responsive, or redox/enzyme-responsive activation systems. Poly(2-ethyl-2-oxazoline)-poly(ε-caprolactone) (PEOz-PCL or PPL) was used as a biocompatible and biodegradable polymer [119]. Since the protonation of tertiary amines on the PEOz backbone facilitates the escape of nuclear endosomes and drug release, it has been used to construct pH-responsive drug carriers [120]. Lan et al. formed PPL-targeted cartilage (C-PPL) by grafting a specific type II collagen-targeting peptide (Coll-II α1 chain-binding peptide-Collb) with the WRYGRL sequence onto PPL. At the same time, PPL was coupled to a peptide substrate specific for the MMP-13 enzyme (H2N-GPLGVRGC-SH) and labeled with a fluorescent dye (Cy5.5). Further, black hole burst-3 (BHQ-3), which can burst Cy5.5 fluorescence, was coupled by an amide reaction to obtain the MMP-13-responsive and pH-sensitive polymer MR-Cy5.5-BHQ-3-PPL (MR-PPL). Finally, cartilage-targeted and OA-specific therapeutic nanoplatforms (MRC-PPL) were obtained by self-assembly of C-PPL and MR-PPL and further used as a carrier to load the traditional Chinese medicine psoralen (PSO) (Figure 9a). Herein, a nanoplatform for the treatment of OA is demonstrated that is activated by the acidic microenvironment and overexpression of the MMP-13 enzyme in OA joints. Incorporated collagen type II-binding peptides facilitate targeting and retention within the joint (Figure 9b). This disease-specific stimulation response strategy improves efficiency and minimizes side effects [121].

Through the enzymatic reaction of fibrinogen and thrombin, Wu et al. designed and constructed in situ nanocomposite hydrogels loaded with KGN and BMSCs. Meanwhile, a ROS-responsive TK liposome was synthesized to load the cartilage formation inducing factor KGN, the biogenic enzyme thrombin, and the ultrasound sensitizer PpIX. Under ultrasound stimulation, TK-based liposomes were disrupted, followed by in situ gelation of fibrinogen and thrombin to fill tissue defects. In addition, the amplification of ROS and sustained release of KGN were achieved by adjusting the ultrasound conditions. More importantly, the level of ROS generated and the amount of KGN released in the in situ nanocomposite hydrogel microenvironment significantly promoted the chondrogenic differentiation of BMSCs and effectively promoted cartilage regeneration in a rat articular cartilage defect model via the Smad5/mTOR signaling pathway [122]. Since the OA microenvironment is characterized by MMP-13 overexpression and weak acidity, Chen et al. designed a new biocompatible cartilage-targeted and MMP-13/pH-responsive ferritin nanocage (CMFn) loaded with anti-inflammatory drugs (hydroxychloroquine, HCQ), called CMFn@HCQ, for OA imaging and therapy (Figure 9c). Two substrates were chemically coupled to the surface of the CFn nanocages, a MMP-13-cleavable specific peptide substrate (H2N-GPLGVRGC-SH) labeled with a fluorescent NIR dye (cy5.5), and a bursting agent for cy5.5 (Black Hole Bursting Agent-3, BHQ3), which enabled synthesized cartilage-targeted and MMP-13-sensitive ferritin (CMFn) nanocages to become “smart” in terms of drug release. CMFn can “turn on” NIR fluorescence specifically for overexpressed MMP-13 to allow real-time fluorescence imaging of the presence of MMP-13 (thus, for early diagnosis and real-time monitoring of the OA locus). When MMP-13 is at its lowest level, such as in normal cartilage or in the presence of a MMP-13 inhibitor, the NIR dye is not released, and therefore, its fluorescence is burst (i.e., turned off) by BHQ3. In the acidic OA joint microenvironment, the nanocage will be dissociated to release HCQ due to acid-induced ferritin catabolism. This smart cartilage-targeted MMP-13/pH dual-stimulation-responsive therapeutic nanoprobe not only provides precise imaging with ultra-high specificity and reversibility but also increases the drug retention time in OA joints (Figure 9d) [123].

## 5. Conclusions and Perspectives

With the intensive development and positive advances in drug delivery platform technologies, a variety of DDSs have been developed for the treatment of OA to prolong the retention of drugs in the joint cavity. Among these DDSs, nanoparticles; liposomes; hydrogels; microspheres with or without capacity in response to endogenous stimuli (redox reactions, pH, enzymes, and temperature); and exogenous stimuli (NIR, magnetic, and ultrasound) not only improve drug solubility and stability but also enhance drug targeting and efficient drug delivery [124]. Though many significant advances have been achieved in preliminary in vivo research (most of them are OA model small animals like mice or rats), there is a need in clinical practice to design stimuli-responsive DDSs with good storage and transport stability and the ability to efficiently release drugs to targets in response to exogenous and endogenous stimuli. In addition, the system can adjust the dosage of medication according to the patient’s needs without the need for frequent injections, which also minimizes side effects [125]. From this point of view, stimulus–response delivery systems are a very interesting and useful method for tuning drug releases. However, stimuli-responsive DDSs also face many challenges. More research is needed to improve the accuracy, precision, and reproducibility of drug deliveries. In addition, the sensitivity to specific stimuli must be higher, as lots of external stimuli (e.g., magnetic fields, light, and heat) can cause damage to healthy tissues [126,127]. Furthermore, when utilizing stimuli-responsive nanomaterials for the delivery of anti-arthritic therapeutics, it is crucial to account for the impact of disease-specific anatomical and physiological barriers (e.g., high-density non-vascular cartilage extracellular matrix) on the bioavailability of nanocarriers within the target joint, in addition to investigating drug release at the targeted site [29,63]. Undoubtably, these issues will be gradually resolved as research in this area continues. Overall, the stimulus-responsive DDS has promising clinical translational prospects and more exploratory prospects for the regeneration and repair of articular cartilage.

## Figures and Tables

**Table 1:** Endogenous stimulus response delivery systems.

Internal Stimulus	Materials	Responsive Shell	Bioactive Agent	Effects	Reference
ROS	Poly (ethylene glycol diacrylate) (PEGDA)-1,2-ethylenedithiol (EDT) copolymer (PEGDA-EDT); reduced graphene oxide (rGO)	PEGDA-EDT	rGO	1. Inhibit the expression of inflammatory cytokines with strong anti-inflammatory effect 2. Upregulation of key antioxidant factors has a strong antioxidant effect	[71]
	Mesoporous silica NPs (MSN) modified with methoxy polyethylene glycol-thioketal (TK), loaded with the small molecule compound oltipraz (OL)	MSN	OL	MSN-OL significantly activated the Nrf2/HO-1 signaling pathway and exhibited better ROS scavenging and stronger antiapoptotic ability to protect the mitochondrial membrane potential of chondrocytes.	[72]
	The NPs consisted of an amphiphilic block copolymer composed of PEG and oxidatively reactive hydrophobic blocks (acrylic monomer of phenylboronic ester and 1,8-naphthalimide fluorescent monomer) encapsulating doxorubicin (DOX)	PEG, phenylboronic ester and naphthalimide	DOX	1. Both copolymer NPs and their degradation products are cytocompatible. 2. ROS stimulated the release of nanoparticle-loaded DOX and allowed polymer degradation to be monitored by scaled fluorescence imaging.	[73]
	Synthesis of nanoparticulate DLNPs with -SeSe- moiety as ROS-responsive component, anti-inflammatory drug dexamethasone (DEX) and chondrogenic differentiation factor chondrogenic derivative -luminescent protein-1 as main pharmacophore	-SeSe-group	DEX, CDMP-1	1. Effectively inhibit the proliferation of activated macrophages, induce macrophage apoptosis, with anti-inflammatory effect, so that BMSCs differentiate into chondrocytes. 2. High concentration of ROS in the joint cavity leads to -SeSe- breakage. The slow release of DEX reduces pain and inflammation.	[74]
	PEG micelles prepared with ROS-sensitive thioketal (TK) and cartilage-targeting peptide (known as TKCP) modified PEG micelles were then encapsulated with DEX to form TKCP@DEX NPs.	TK	DEX	1. Nanoprobes can intelligently “turn on” in response to excessive ROS and “turn off” in normal joints. 2. TKCP@DEX can effectively respond to ROS and slow-release DEX to significantly reduce cartilage damage in OA joints.	[75]
	Pinacol borate, PEG and naphthylamide monomers with encapsulated hydrophobic curcumin	polymethacrylate	Cur	Both in vitro and in vivo studies validated real-time visualization of drug release and ROS clearance, as well as therapeutic efficacy in OA.	[76]
pH	HA-modified metal–organic frameworks (MOFs) system loaded with anti-inflammatory protocatechuic acid (PCA)	MOFs	PCA	1. Significantly reduced IL-1β-induced synovial inflammation in chondrocytes and OA joints. 2. Downregulates the expression of OA inflammatory markers and promotes the expression of cartilage-specific genes.	[77]
	Modified mesoporous silica NPs (MSNs) and polyacrylic acid (PAA), loaded with andrographolide (AG)	PAA	AG	Shows stronger anti-arthritic efficacy and cartilage protection, as evidenced by lower expression of inflammatory factors and better prevention of proteoglycan loss.	[78]
	Cyclic brushed amphiphilic polymer (CB) with SBMA and DMAEMA as brushes and cyclic polymer (c-P(HEMA)) as core template, loaded with hydrophobic curcumin (Cur) and hydrophilic loxoprofen (LXP)	DMAEMA	Cur, LXP	1. Hydrophilic and hydrophobic anti-inflammatory drugs can be co-loaded with higher drug loading efficiency. 2. With over-lubrication, sequence-controlled release and anti-inflammatory effects, it can effectively treat OA.	[79]
	PLGA, ammonium bicarbonate (NH_4_HCO_3_), HA	NH_4_HCO_3_	HA	1. The NPs are non-toxic to chondrocytes and have no negative impact on joints. 2. Exhibits extracellular burst release behavior and higher chondrocyte viability.	[80]
	Hollow mesoporous silica NPs (HMSN), chitosan (Cs) as coating, loaded with celastrol (CSL)	Cs	CSL	1. High biocompatibility for intra-articular injection. 2. Downregulate the expression of inflammatory factors, improve joint surface erosion and joint effusion.	[81]
Enzyme	Triglyceride monostearate (TG-18), corticosteroid triamcinolone acetonide (TA)	TG-18	TA	Provide the optimal amount of therapeutic medication when needed, thereby maximizing the therapeutic effect and prolonging the duration of the therapeutic effect.	[82]
	Schiff base cross-linking between oxidized hyaluronic acid (OHA) and type I collagen to form collagen-based hydrogels (Col-OHA); dexamethasone sodium phosphate (DSP)-loaded	Col-OHA	DSP	It has a significant inhibitory effect on the production of synovial inflammatory cytokines and provides effective and sustained relief of OA symptoms.	[83]
Temperature	Poly (organophosphorus nitrile) NPs (TePN), encapsulated tretinoin (TCA)	TePN	TCA	OA is treated by inhibiting MMP expression in cartilage by decreasing pro-inflammatory cytokine expression and increasing anti-inflammatory cytokine expression.	[84]
	Poloxamer 407 (P407), HA, copper nanodots (Cu NDs), platelet rich plasma (PRP)	P407	Cu NDs, HA, PRP	1. It can remove RON in the joint microenvironment and block the destructive effect of RONS on chondrocytes 2. It can reverse the M1 polarization of macrophages and promote the production of M2 macrophages.	[85]
	Poly(ε-caprolactone-propionate)-b-poly (ethylene glycol)-b-poly(ε-caprolactone-propionate) (PCLA-PEG-PCLA) triblock copolymer, flurbiprofen	PCLA-PEG-PCLA	flurbiprofen	1. Intra-articular administration can effectively prolong the analgesic time. 2. Significantly reduce the inflammatory response of rat OA model by downregulating the expression of inflammatory factors.	[86]
	HA, Pluronic F-127, piroxicam (PX)	Pluronic F-127	PX	It has excellent mechanical strength and also induces sustained drug release behavior.	[87]

**Table 2:** Exogenous stimulus response delivery systems.

External Stimulus	Materials	Responsive Shell	Bioactive Agent	Effects	Reference
NIR	Metal Organic Framework (MOF) modified mesoporous polydopamine (MPDA), collagen II targeting peptide (WYRGRL), loaded with rapamycin (Rap) and bilirubin (Br)	PDA	Rap, Br	1. With excellent near infrared laser stimulation responsive drug release effect. 2. Has excellent MR imaging properties to monitor its in vivo therapeutic effects. 3. Enhanced energy metabolism of chondrocytes, further rescued apoptosis in vitro and inhibited cartilage degeneration in vivo.	[101]
	molybdenum (Mo)-based polyoxometalate clusters (POM)	POM	-	1. It has excellent antioxidant activity, biological safety and good anti-inflammatory effect. 2. Effectively alleviate the symptoms of OA mice, prevent cartilage erosion, reduce inflammatory cytokines, and reduce articular cartilage decomposition and metabolism proteases.	[102]
	Preparation of HSC hydrogels by mixing HA-thiourea (NCSN) solution with Cu^2+^	NCSN, Cu^2+^	NCSN	Effectively promotes chondrocyte anabolism while reducing IL-1β-induced catabolism and inflammation.	[103]
	Mitochondria-targeted and sod-mimicking Mn_3_O_4_@PDA@Pd-SS31 nanoenzymes	PDA	Pd, Mn_3_O_4_	1. It has good biocompatibility and photodynamic effect. 2. Effective removal of ROS in mitochondria, thus improving the oxidative stress microenvironment.	[104]
	Neutrophil erythrocyte hybrid membrane-encapsulated dexamethasone sodium phosphate (Dexp) loaded hollow copper sulf ide NPs (D-CuS@NR NPs)	CuS	Dexp	1. It has excellent photothermal conversion ability, drug release behavior control and good cytocompatibility. 2. It can target the inflamed joint parts with stronger anti-inflammatory effect. 3. After NIR treatment can effectively inhibit cartilage degeneration through photothermal therapy and downregulation of synovial inflammation.	[105]
Magnetic	KGN-loaded magnetic NPs (KGN-MNPs) were synthesized by encapsulating KGN on the surface of iron oxide NPs using poly(propylene lactone) (PLA)	MNP	KGN	1. Significantly increased the retention of KGN in MSCs and improved the utilization of KGN. 2. Inhibited the generation of inflammation, induced the chondrogenic differentiation of MSCs and can prevent articular cartilage degeneration.	[106]
	Mesoporous polydopamine NPs (DAMM NPs) doped with arginine and manganese (Mn) ions, loaded with dexamethasone (DEX)	DAMM NP	DEX	1. Prolonged DEX release can directly ameliorate OA progression by inhibiting macrophage-induced synovial inflammation. 2. It can directly reduce ROS-induced chondrocyte apoptosis. 3. Displaying magnetic resonance imaging (MRI) sensitive signals, thus enabling real-time visualization of damaged articular cartilage.	[107]
	Modified natural polysaccharide hyaluronic acid (HAMA) and chondroitin sulphate (CSMA), loaded with magnetic NPs (MPM) and the anti-inflammatory drug DS	Fe_3_O_4_@MgSiO_3_	DS	1. It is mono-disperse, porous and facilitates slow drug release, and its magnetic properties give it controlled drug delivery. 2. Can effectively alleviate cartilage degradation in OA rats	[108]
Ultrasonic	Pluronic^®^ F-127, HA, gelatin, loaded with hydrocortisone	Pluronic	hydrocortisone	1. Has thermosensitive properties and is capable of sustaining drug release. 2. Has controlled on-demand drug release and provides higher hydrocortisone concentrations in OA. 3. Possesses ultrasound, thermal response and stability.	[45]
	Carboxymethyl chitosan oxidized chondroitin sulphate (CMC-OCS) hydrogel-embedded KGN loaded PLGA MPs (MPs@KGN) (CMC-OCS@MPs@KGN)	PLGA	KGN	1. Exhibits faster gelation, lower swelling ratio and lower in vitro degradation. 2. Has the ability to increase COL-2 synthesis, which facilitates cartilage repair. 3. Can respond to ultrasound by controlled KGN burst release.	[109]

## Data Availability

Not applicable.
